# Supplementary material for: Both IgM and IgG Antibodies against Polyethylene Glycol Can Alter the Biological Activity of Methoxy Polyethylene Glycol-Epoetin Beta in Mice
Source: Pharmaceutics. 2019 Dec 21;12(1):15. doi: 10.3390/pharmaceutics12010015 (PMC7022322; doi:10.3390/pharmaceutics12010015)
Supplement: Supplementary file 1 [file pharmaceutics-12-00015-s001.pdf]

pharmaceutics

Supplementary Materials: Both IgM and IgG Antibodies against Polyethylene Glycol Can Alter the Biological Activity of Methoxy Polyethylene Glycol-Epoetin Beta in Mice

Tien-Ching Chang , Bing-Mae Chen, Wen-Wei Lin, Pei-Hua Yu, Yi-Wen Chiu, Yuan-Tsong Chen, Jer-Yuarn Wu, Tian-Lu Cheng, Daw-Yang Hwang and Steve Roffler

Table 1. Sources of antibodies.

Antibody	Source	Catalog No.	Assay
6.3 anti-PEG IgG	Academia Sinica (Taipei, Taiwan)	6.3-PABG-A	in all studies
AGP4 anti-PEG IgM	Academia Sinica	AGP4-PABM-A	in all studies
Human IgG_1	Abcam (Cambridge, UK)	Ab90283	Human IgG_1 negative control antibody
Human IgM	Rockland (Limerick, PA)	009-0107-0001	Human IgM negative control antibody
Goat IgG anti-erythropoietin	R&D system (Minneapolis, MN)	AF959	Neutralization assay and immunoblotting
Peroxidase-conjugated affinipure goat $F(ab')_2$ anti-human IgG Fcγ	Jackson ImmunoResearch (West Grove, PA)	109-036-098	Second antibody in anti-PEG IgG ELISA
Peroxidase-conjugated affinipure rabbit anti-human IgM $Fc_{5\mu}$	Jackson ImmunoResearch	309-035-095	Second antibody in anti-PEG IgM ELISA
Peroxidase-conjugated affinipure donkey anti-mouse IgG (H+L)	Jackson ImmunoResearch	715-035-150	Second antibody in anti-PEG IgG ELISA and immunoblotting
Peroxidase-conjugated affinipure goat anti-mouse IgM μ chain	Jackson ImmunoResearch	115-035-020	Second antibody in anti-PEG IgM ELISA and immunoblotting
Peroxidase-conjugated goat anti-mouse IgG $F(ab')_2$	Cappel™, Organon Teknika Corporation (Durham, NC)	55553	Second antibody in anti-PEG IgG $F(ab')_2$ ELISA
Peroxidase-conjugated goat anti-mouse IgG Fc	Cappel™, Organon Teknika Corporation	55554	Second antibody in anti-PEG IgG $F(ab')_2$ ELISA

Table S2. Concentrations of anti-PEG antibodies in human serum samples.

Sample name	Anti-PEG IgM (μg mL^{-1})	Anti-PEG IgG (μg mL^{-1})
hM1	4.93	0
hM2	8.01	0
hG1	0	15.0
hG2	0	34.8
hMG1	6.66	4.65
hMG2	2.52	5.16

Samples were from normal Taiwanese donors.

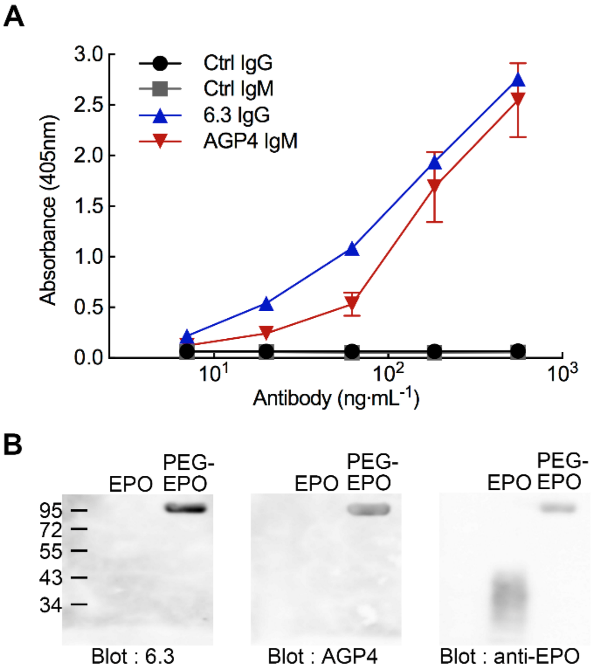

Figure S1. Mouse anti-PEG AGP4 IgM and 6.3 IgG antibodies bind to PEG-EPO. (**A**) Serial dilutions of AGP4 and 6.3 monoclonal antibodies were assayed for binding to PEG-EPO in 96-well plates using peroxidase-conjugated affinipure anti-mouse IgM or IgG specific secondary antibodies. Control IgM (Ctrl IgM) and control IgG (Ctrl IgG) are negative control mouse IgM and IgG antibodies, respectively. (bars, SD; n = 2). (**B**) Recombinant human erythropoietin (EPO) and PEG-EPO were immunoblotted with 6.3, AGP4, or anti-EPO and specific secondary antibodies.

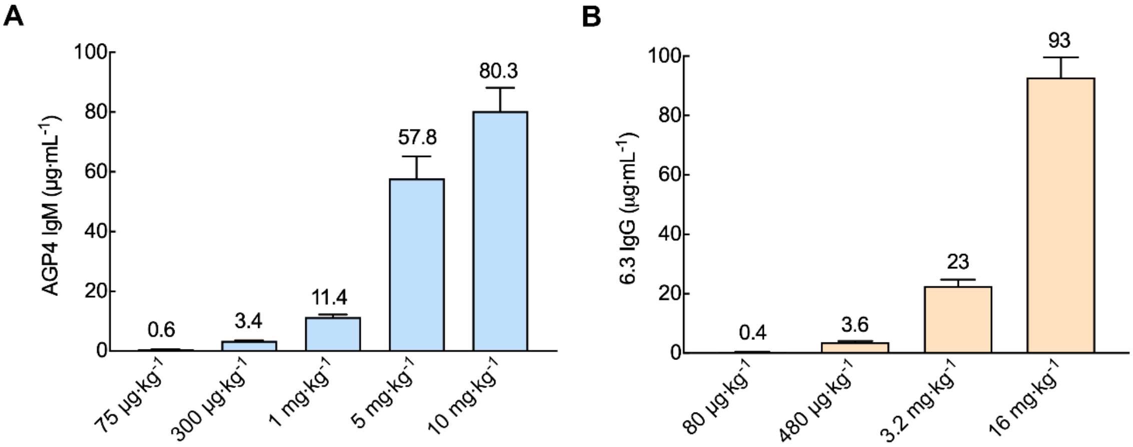

Figure S2. Measurement of anti-PEG antibodies in serum at 24 h after injection. BALB/c mice were intravenously injected with different doses of AGP4 (**A**) or 6.3 (**B**) antibodies. At 24 h after injection, serum samples were collected and the concentrations of anti-PEG antibodies were measured by direct ELISA on PEG-coated plates using peroxidase-conjugated affinipure anti-mouse IgM or IgG specific secondary antibodies. (bars, SD; n = 4)

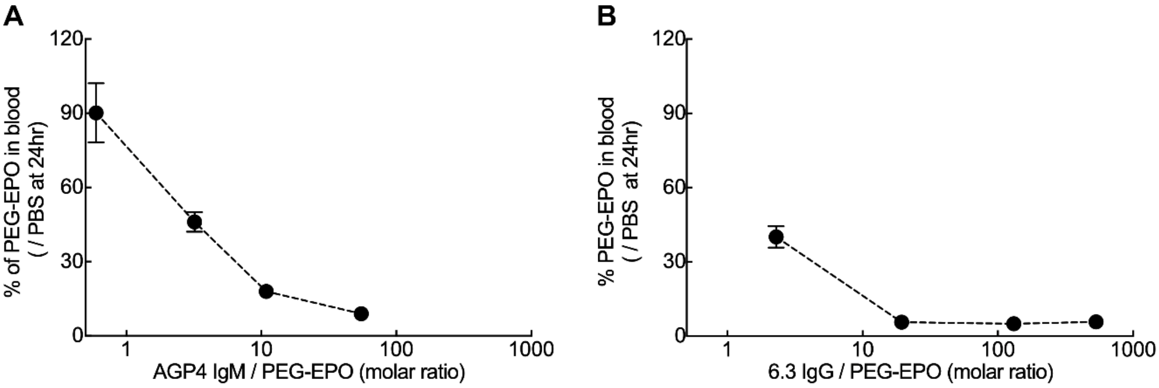

Figure S3. Anti-PEG antibodies accelerate clearance of PEG-EPO in mice. Female BALB/c mice were pre-injected with different doses of mouse anti-PEG AGP4 IgM or 6.3 IgG 24 h before intravenously injection of 5 μg kg^{-1} ^{125}I-PEG-EPO. Radioactivity was measured in plasma samples collected at 30 min, 2 h, 6 h and 24 h. Percentages of PEG-EPO remaining in the circulation at different molar ratios of AGP4 (**A**) or 6.3 (**B**) are shown. (n = 4).

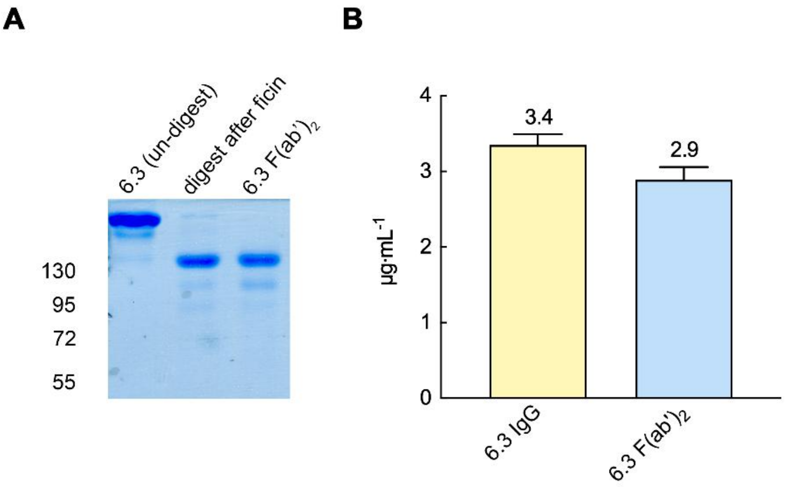

Figure S4. Characterization of 6.3 F(ab′)$_2$. (**A**) Non-reducing SDS-PAGE electrophoresis of 6.3 IgG and 6.3 F(ab′)$_2$ fragments. (**B**) Concentrations of 6.3 IgG and 6.3 F(ab′)$_2$ in mouse serum 1 h after i.v. injection (bars, SD; n = 4).
